# Editorial: Molecular Advances in Diagnosis and Treatment of CNS Tumors

**DOI:** 10.3389/fonc.2020.590293

**Published:** 2020-09-25

**Authors:** Zhao-Hui Zhang, Ming-Tseh Lin, Liam Chen

**Affiliations:** ^1^Department of Neurology, Renmin Hospital of Wuhan University, Wuhan, China; ^2^Department of Pathology, Johns Hopkins University School of Medicine, Baltimore, MD, United States; ^3^Department of Laboratory Medicine and Pathology, University of Minnesota Medical School, Minneapolis, MN, United States

**Keywords:** brain tumor, glioblastoma, glioma, astrocytic, molecular biomarker, therapy

Recent advances in the field of molecular pathology and the publication of the revised fourth edition of the WHO Classification of central nervous system (CNS) tumors have significantly reshaped the approach to both diagnosis and therapy of brain tumors (1). Due to rapid development of next generation sequencing techniques, molecular-genetic analysis has now become an integral part of modern surgical neuropathology. Current diagnosis of CNS tumors routinely combines the results from histologic and immunohistochemical examinations of microscopic slides with the key DNA/RNA genetic changes identified in the molecular pathology testing. This has led to a substantial reclassification of various brain and spinal cord tumors, including the introduction of new neoplastic entities and removal of others. Involvement of key tumor suppressor genes and oncogenes in brain tumor development has been known for decades, but recent studies have highlighted many novel genetic variations occurring in adult and pediatric brain tumors. These emerging discoveries further emphasize the importance of identifying state-of-the-art molecular signatures for diagnosing CNS malignancy and development of novel targeted therapies. In particular, understanding the molecular landscapes of pediatric high grade astrocytic tumors and embryonal tumors, and capitalizing on immunotherapy which may have the power to revolutionize brain tumor treatment, are a few of the many challenges facing this field today.

This Research Topic entitled “Molecular Advances in Diagnosis and Treatment of CNS Tumors” includes 22 original research articles and 3 review articles that cover several important themes:

**Glioblastoma (GBM) is the most common and devastating primary brain tumor in adults**. It is therefore essential to identify novel and effective biomarkers or risk signatures for GBM patients. Wang et al. examined differentially expressed genes between GBM and low-grade glioma (LGG) and selected five genes (DES, RANBP17, CLEC5A, HOXC11, and POSTN) to construct a risk signature to independently predict the outcome of GBM patients, as well as stratified by radio-chemotherapy, isocitrate dehydrogenase 1 (IDH1) and O6-methylguanine-DNA methyltransferase (MGMT) promoter status. Zhang et al. evaluated the expression level of integrin beta 5 (ITGB5) and the relationship of its elevated expression with glioma progression and poor survival in GBM patients. It appears ITGB5 plays important regulatory roles in angiogenesis and the immune response, and is required for invasion and migration of neoplastic cells and endothelial proliferation in GBM. Zusman et al. discussed how harvesting GBM tissue using traditional surgical approach and the automated resection NICO Myriad™ system may impact the translational research value of the sample. Their study further supports the need to harvest and analyze multiple specimens for each tumor, in order to capture the genomic diversity and maximize the benefits of molecularly-based therapeutics. Zhang et al. demonstrated that Forkhead Box P2 (FOXP2) was the target protein of miR-9-5p. In addition, high expression of miR-9-5p and low expression of FOXP2 were related to better outcome in GBM patients, whereas down regulated FOXP2 expression was capable of inhibiting glioma proliferation through cell cycle arrest. Liu et al. determined the candidate genes that may function as biomarkers to further distinguish patients with IDH-wildtype GBM. The investigators developed a seven-gene-based signature, which allocated each patient to a risk group (low or high). Subsequent bioinformatics analysis predicted that the seven-gene signature was involved in the immune response, inflammatory response, cell adhesion, and apoptotic process. Marchi et al. attempted to correlate the biomolecular aspects of MGMT methylation status in relation to the maximal surgical extent of resection. Interestingly, a positive prognostic value exists only in case of the presence of residual tumor tissue. Dent et al. explored whether a multiple sclerosis drug, Fingolimod would synergize with dimethyl fumarate and its plasma breakdown product MMF to kill GBM neoplastic cells. Indeed, the data demonstrated that the above combination produced reactive oxygen species and killed tumor cells more effectively via death receptor signaling and autophagy induction. Hu et al. conducted a systematic analysis of survival-associated alternative splicing event. The nomogram with age, pharmaceutical and radiation therapy, alternate donor site, and exon skip signatures provided excellent prognostic predictive value.

**Treatment effectiveness and overall prognosis for glioma patients depend heavily on the genetic and epigenetic factors in each individual tumor**. Gates et al. discovered that primary brain tumors are genetically heterogeneous, and the physical distance within a given glioma positively correlates to genomic distance in number of genes, copy number variations, and methylation profiles. They further derived quantitative linear relationships between physical and genomic distances. Su et al. showed that γKlotho (also known as LCTL) is highly expressed in gliomas epigenetically and its expression is significantly associated with high tumor aggressiveness and poor outcomes for glioma patients. Mechanistically, LCTL might play an important immunosuppressive role via FGF signaling in glioma. Yang et al. performed weighted gene co-expression network analysis in a large public database of glioma samples. The derived brown co-expression module and the biomarker TNFRSF1A were strongly related to glioma grading. Furthermore upregulated TNFRSF1A was tightly associated with clinical features. Zhang et al. systematically analyzed the relationship between methyltransferase-related gene expression profiles and clinical outcomes in glioma patients and identified a novel methyltransferase-related risk signature for predicting the prognosis of gliomas.

**Recently non-coding types of RNA have been shown to play a vital role in glioma tumorigenesis**. Jin et al. characterized a novel non-coding RNA, lipocalin-2-derived circular RNA, in glioma tumorigenesis. The investigators demonstrated that it facilitated glioma progression by sponging miR-661 to increase RAB3D expression. Similarly, Zheng et al. characterized non-coding competitive RNA networks as alternative therapeutic targets in the treatment of GBM. Sun et al. explored the expression profiles and potential relationship between long non-coding RNAs (lncRNAs) and mRNAs in glioma patients. Both lncRNAs and mRNAs exhibited dynamic differential expression profiles, consistent with their roles in critical biological processes and pathways associated with tumor pathogenesis.

**Several manuscripts cover some of the most fascinating developments in the field**. Zhang et al. conducted a meta-analysis to evaluate the prognostic role of connexin protein Cx43 in glioma. The results showed that Cx43 expression was a clearly negative factor with tumor grades and beneficial for survival time, offering evidence that Cx43 is generally a tumor suppressor. Deng et al. explored the influence of IDH1 mutation on the immune microenvironment and developed an IDH1-associated immune prognostic signature to help classify LGG patients into subgroups with distinct outcomes and immunophenotypes. Liu et al. discussed the correlations of soluble PD-L1 (sPD-L1) with clinical features in brain tumors and assessed its diagnostic value in gliomas. Both serum and CSF sPD-L1 showed significant value, but serum sPD-L1 rather than blood-based inflammatory markers had the best diagnostic performance in the diagnosis and stratification of glioma. In addition, a descending trend in the level of serum sPD-L1 was observed in postoperative patients. Hung et al. studied the important question of glioma stem-like cells contributing to drug resistance and tumor recurrence. Their study suggests that a sonic hedgehog (Shh) inhibitor could induce autophagy of CD133+ GSCs through mTOR independent pathway. Therefore, targeting the Shh signal pathway may overcome chemoresistance and provide a therapeutic strategy for patients with malignant gliomas.

**Informative Review Articles:** Tang et al. reviewed the advantages and possible limitations of mRNA-based gene therapy including the *in vitro* synthesis of mRNA, the feasible methods for synthetic mRNA delivery and clinical therapeutic prospects of mRNA-based gene therapy for glioblastoma. Yu et al. reviewed the regulation of MGMT expression and its role in chemotherapy, especially in glioma. Targeting MGMT seems to be a promising approach to overcome chemoresistance. Hu et al. reviewed the relationship between ferroptosis, a new type of cell death, and temozolomide (TMZ) resistance. Importantly, targeted ferroptosis can be used to reverse TMZ resistance.

In summary, management of CNS tumor patients has undergone a molecular revolution driven by the development of high throughput molecular techniques. Molecular testing has become an essential part for the optimal CNS tumor patient workup. At the current stage, a combination of FISH, copy number array, NGS panel and genome-wide methylation profiling can be used to detect molecular alterations in order to provide the best possible patient care. It is true that our ability of amassing molecular data currently surpasses our ability to utilize this information for treatment; however, it is clear that informative molecular biomarkers will guide future clinical trials and lead to the development of new therapeutic strategies.

## Author Contributions

Z-HZ, M-TL, and LC are the coeditors for this Research Topic. All authors contributed to the article and approved the submitted version.

## Conflict of Interest

The authors declare that the research was conducted in the absence of any commercial or financial relationships that could be construed as a potential conflict of interest.
